# Cetylpyridinium bromide monohydrate: localization of H atoms

**DOI:** 10.1107/S2414314625009915

**Published:** 2025-11-14

**Authors:** Kina Muller, Eric Cyriel Hosten, Richard Betz

**Affiliations:** a Nelson Mandela University, Summerstrand Campus, Department of Chemistry, University Way, Summerstrand, PO Box 77000, Port Elizabeth, 6031, South Africa; Goethe-Universität Frankfurt, Germany

**Keywords:** crystal structure, C—H⋯Br contacts, C—H⋯O contacts

## Abstract

The title compound, C_21_H_38_N^+^·Br^−^·H_2_O, is the bromide salt of a quarternary pyridinium cation bearing a hexa­decyl chain on the nitro­gen atom. One mol­ecule of solvent water is present in the asymmetric unit. Classical hydrogen bonds of the O—H⋯Br type are observed next to C—H⋯Br and C—H⋯O contacts that connect the entities of the title compound to sheets parallel to the *ab* plane.

## Graphical abstract

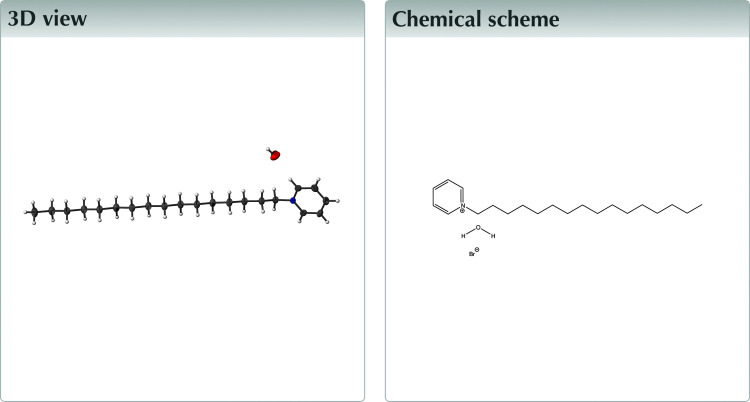

## Structure description

The effect of size and steric pretence of large ions on the chemical and spectroscopic properties of compounds have been a focus of research for many decades. Many effects can be attributed to the spatial requirements of counter-ions such as the glass-transition temperature in ionomers (Enokida *et al.*, 2020 ▸) or surfactant-modifying properties (Oh & Shah, 1993 ▸), the charge transfer in radical ions (Piotrowiak & Miller, 1993 ▸) and polymer-modified electrodes (Ma­thias & Haas, 1993 ▸) as well as the structural and vibrational spectroscopic behaviour of DNA building blocks (Minguirbara *et al.*, 2020 ▸). In addition, it has been found that selecting an adequate size of counter-ions to crystallize ionic compounds can be crucial for the isolation of crystalline reaction products of high quality, the latter having been confirmed and reviewed on many occasions (Roof & Kolis, 1993 ▸), with bis­(tri­phenyl­phosphine)iminium being a good example for the successful crystallization of bulky transition-metal coordination compounds (McNicholas *et al.*, 2023 ▸). In a continuation of our inter­est in metrical features of large cations (Muller *et al.*, 2021*a* ▸,*b* ▸,*c* ▸,*d* ▸,*e* ▸,*f* ▸,*g* ▸; Hosten *et al.*, 2012 ▸; Hosten *et al.*, 2015*a* ▸,*b* ▸; Hosten & Betz, 2024 ▸; Schoultz *et al.*, 2015 ▸), the structure of the title compound has been determined. While the latter has been reported previously (Ballirano *et al.*, 1998 ▸), no hydrogen-atom positions were determined, which precludes discussions of inter- and intra­molecular inter­actions on a comparative basis in the envisioned ionic target compounds. The mol­ecular and crystal structures of co-crystallizates of the title compound have been reported earlier (Iimura *et al.*, 2002 ▸), as were the structures of the hydrated and anhydrous quinolinium equivalents (Wieckowski *et al.*, 2024 ▸). Furthermore, the mol­ecular and crystal structures of two compounds featuring the title compound’s cationic residue as leitmotif are apparent in the literature (Dalcanale *et al.*, 2021 ▸; Lu *et al.*, 2011 ▸). Cetylpyridinium bromide (CPB) as such has been employed extensively in germicidal applications, as well as in enzyme studies where CPB was used for polymerization, protein folding, gene delivery and, eventually, as a drug delivery agent in pharmaceuticals (Verma *et al.*, 2015 ▸; Ali *et al.*, 2023 ▸).

**Figure 1:**
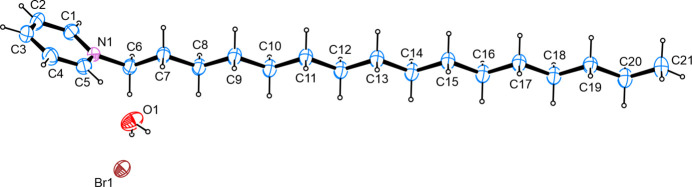
The mol­ecular structure of the title compound, with atom labels and anisotropic displacement ellipsoids (drawn at the 50% probability level).

The structure solution shows the presence of an alkyl­ated derivative of pyridine with the nitro­gen atom bearing a hexa­decyl chain. The positive charge of this pyridinium cation is counterbalanced by a bromide anion. Furthermore, a mol­ecule of water is present in the asymmetric unit. The alkyl chain adopts an ideal zigzag conformation with all its carbon atoms being co-planar, and the largest deviation from the least-squares plane as defined by these carbon atoms measured at 0.026 (2) Å. The latter plane inter­sects with the least-squares plane as defined by the non-hydrogen atoms of the aromatic system at an angle of 60.30 (9)°. Intra­cyclic angles cover a range from 118.72 (17)–120.62 (17)° with the smallest angle on the carbon atom opposite from the pnicogen atom and the largest angle on one of the intra­cyclic carbon atoms directly bonded to the nitro­gen atom (Fig. 1 ▸). Bond lengths are in good agreement with other alkyl­ated pyridinium derivatives whose metrical parameters have been deposited with the Cambridge Structural Database (Groom *et al.*, 2016 ▸).

**Figure 2:**
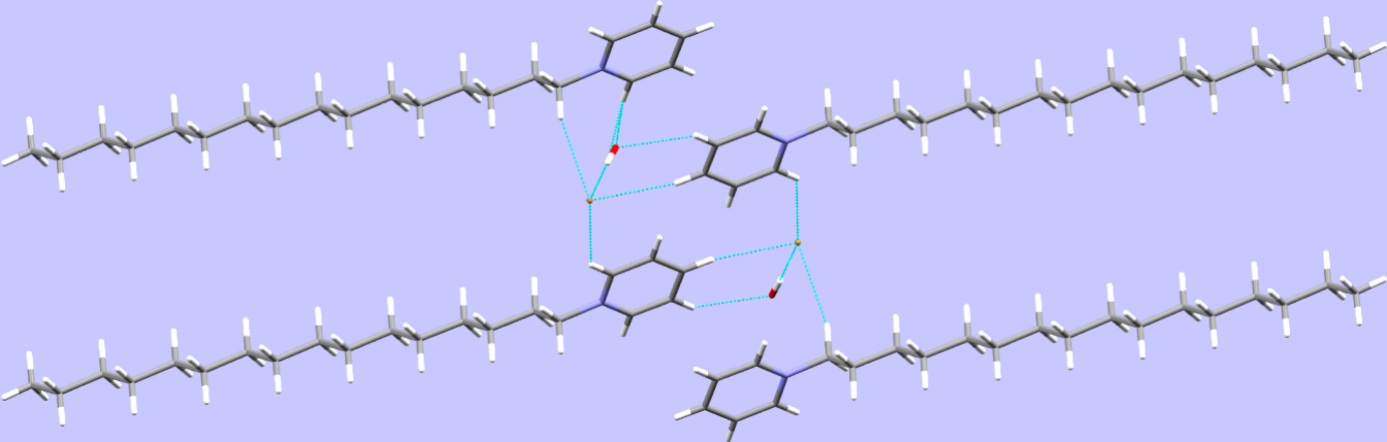
Selected inter­molecular contacts, viewed along [010].

In the crystal structure, classical hydrogen bonds of the O—H⋯Br type (Table 1 ▸) are observed next to C—H⋯O as well as C—H⋯Br contacts whose range falls by more than 0.1 Å below the sum of van der Waals radii of the atoms participating in them. The C—H⋯O contacts are established by one of the aromatic CH groups in *ortho* position to the nitro­gen atom as well as the adjacent aromatic CH group in the *meta* position to the pnicogen atom while the C—H⋯Br contacts are observed between the second aromatic CH group in an *ortho* position to the nitro­gen atom and the aromatic CH group in a *para* position as well as one of the hydrogen atoms of the methyl­ene group directly bonded to the intra­cyclic heteroatom (Fig. 2 ▸). In total, the entities of the asymmetric unit are connected into a three-dimensional network. In terms of graph-set analysis (Etter *et al.*, 1990 ▸; Bernstein *et al.*, 1995 ▸), the classical hydrogen bonds require a *DD* descriptor on the unary level while the C—H⋯O and C—H⋯Br contacts necessitate a *DDD* descriptor on the same level. π-Stacking is not a prominent feature in the crystal structure of the title compound with the shortest inter­centroid distance measured at 4.6493 (11) Å.

## Synthesis and crystallization

The compound was obtained commercially (Fluka). Crystals suitable for the diffraction study were obtained upon recrystallization from boiling water.

## Refinement

Crystal data, data collection and structure refinement details are summarized in Table 2 ▸. The carbon-bound H atoms were placed in calculated positions (C—H = 0.95 Å for the aromatic carbon atoms and C—H = 0.99 Å for the methyl­ene groups) and were included in the refinement in the riding model approximation, with *U*_iso_(H) set to 1.2*U*_eq_(C). The H atoms of the methyl group were allowed to rotate with a fixed angle around the C—C bond to best fit the experimental electron density [HFIX 137 in the *SHELX* program suite (Sheldrick, 2015)], with *U*_iso_(H) set to 1.5*U*_eq_(C) and with C—H 0.98 Å. The hydrogen atoms of the water mol­ecule were located on a difference-Fourier map and refined freely with the O—H distances restrained to 0.84 (1) Å and the H⋯H distance restrained to 1.34 (2) Å.

## Supplementary Material

Crystal structure: contains datablock(s) I. DOI: 10.1107/S2414314625009915/bt4188sup1.cif

Structure factors: contains datablock(s) I. DOI: 10.1107/S2414314625009915/bt4188Isup2.hkl

Supporting information file. DOI: 10.1107/S2414314625009915/bt4188Isup3.cml

CCDC reference: 2501304

Additional supporting information:  crystallographic information; 3D view; checkCIF report

## Figures and Tables

**Table 1:** Hydrogen-bond geometry (Å, °)

*D*—H⋯*A*	*D*—H	H⋯*A*	*D*⋯*A*	*D*—H⋯*A*
O1—H1*A*⋯Br1^i^	0.84 (1)	2.49 (1)	3.3254 (14)	177 (2)
O1—H1*B*⋯Br1	0.84 (1)	2.49 (1)	3.3227 (16)	171 (2)
C1—H1⋯Br1^ii^	0.95	2.87	3.5890 (17)	133
C3—H3⋯Br1^iii^	0.95	2.85	3.7797 (18)	168
C4—H4⋯O1^iv^	0.95	2.59	3.362 (3)	138
C5—H5⋯O1	0.95	2.28	3.218 (2)	171
C6—H6*B*⋯Br1	0.99	2.84	3.7702 (18)	156

Symmetry codes: (i) 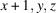; (ii) 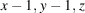; (iii) 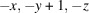; (iv) 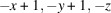.

**Table 2:** Experimental details

Crystal data	
Chemical formula	C_21_H_38_N^+^·Br^−^·H_2_O
*M*_r_	402.45
Crystal system, space group	Triclinic, *P* 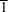
Temperature (K)	200
*a*, *b*, *c* (Å)	5.5164 (2), 7.5140 (2), 27.3705 (9)
α, β, γ (°)	94.420 (1), 95.059 (1), 100.723 (1)
*V* (Å^3^)	1105.28 (6)
*Z*	2
Radiation type	Mo *K*α
μ (mm^−1^)	1.87
Crystal size (mm)	0.44 × 0.29 × 0.03

Data collection	
Diffractometer	Bruker APEXII CCD
Absorption correction	Multi-scan (*SADABS*; Krause *et al.*, 2015 ▸)
*T*_min_, *T*_max_	0.466, 0.511
No. of measured, independent and observed [*I* > 2σ(*I*)] reflections	49450, 4490, 4131
*R*_int_	0.040
(sin θ/λ)_max_ (Å^−1^)	0.625

Refinement	
*R*[*F*^2^ > 2σ(*F*^2^)], *wR*(*F*^2^), *S*	0.027, 0.062, 1.13
No. of reflections	4490
No. of parameters	226
No. of restraints	3
H-atom treatment	H atoms treated by a mixture of independent and constrained refinement
Δρ_max_, Δρ_min_ (e Å^−3^)	0.33, −0.35

Computer programs: *APEX2* and *SAINT* (Bruker, 2014 ▸), *SHELXS97* (Sheldrick 2008 ▸), *ORTEP-3 for Windows* (Farrugia, 2012 ▸), *Mercury* (Macrae *et al.*, 2020 ▸), *SHELXL2019/3* (Sheldrick, 2015 ▸) and *PLATON* (Spek, 2020 ▸).

## full crystallographic data

### 1-Hexadecylpyridin-1-ium bromide monohydrate Crystal data

**Table:**

C_21_H_38_N^+^·Br^−^·H_2_O	*Z* = 2
*M**_r_* = 402.45	*F*(000) = 432
Triclinic, *P*1	*D*_x_ = 1.209 Mg m^−^^3^
*a* = 5.5164 (2) Å	Mo *K*α radiation, λ = 0.71073 Å
*b* = 7.5140 (2) Å	Cell parameters from 9771 reflections
*c* = 27.3705 (9) Å	θ = 2.3–26.3°
α = 94.420 (1)°	µ = 1.87 mm^−^^1^
β = 95.059 (1)°	*T* = 200 K
γ = 100.723 (1)°	Platelet, colourless
*V* = 1105.28 (6) Å^3^	0.44 × 0.29 × 0.03 mm

### 1-Hexadecylpyridin-1-ium bromide monohydrate Data collection

**Table:**

Bruker APEXII CCD diffractometer	4490 independent reflections
Radiation source: sealed tube	4131 reflections with *I* > 2σ(*I*)
Graphite monochromator	*R*_int_ = 0.040
φ and ω scans	θ_max_ = 26.4°, θ_min_ = 2.3°
Absorption correction: multi-scan (SADABS; Krause *et al.*, 2015)	*h* = −6→6
*T*_min_ = 0.466, *T*_max_ = 0.511	*k* = −9→9
49450 measured reflections	*l* = −34→34

### 1-Hexadecylpyridin-1-ium bromide monohydrate Refinement

**Table:**

Refinement on *F*^2^	Primary atom site location: structure-invariant direct methods
Least-squares matrix: full	Secondary atom site location: difference Fourier map
*R*[*F*^2^ > 2σ(*F*^2^)] = 0.027	Hydrogen site location: mixed
*wR*(*F*^2^) = 0.062	H atoms treated by a mixture of independent and constrained refinement
*S* = 1.13	*w* = 1/[σ^2^(*F*_o_^2^) + (0.0194*P*)^2^ + 0.5567*P*] where *P* = (*F*_o_^2^ + 2*F*_c_^2^)/3
4490 reflections	(Δ/σ)_max_ = 0.001
226 parameters	Δρ_max_ = 0.33 e Å^−^^3^
3 restraints	Δρ_min_ = −0.35 e Å^−^^3^

### 1-Hexadecylpyridin-1-ium bromide monohydrate Special details

**Table:**

Geometry. All esds (except the esd in the dihedral angle between two l.s. planes) are estimated using the full covariance matrix. The cell esds are taken into account individually in the estimation of esds in distances, angles and torsion angles; correlations between esds in cell parameters are only used when they are defined by crystal symmetry. An approximate (isotropic) treatment of cell esds is used for estimating esds involving l.s. planes.

### 1-Hexadecylpyridin-1-ium bromide monohydrate Fractional atomic coordinates and isotropic or equivalent isotropic displacement parameters (Å^2^)

**Table:**

	*x*	*y*	*z*	*U*_iso_*/*U*_eq_	
Br1	0.28243 (3)	0.95301 (3)	0.12071 (2)	0.03558 (7)	
O1	0.7091 (3)	0.7168 (2)	0.09060 (6)	0.0504 (4)	
N1	0.0383 (2)	0.38072 (18)	0.10659 (5)	0.0266 (3)	
C1	−0.1706 (3)	0.2569 (2)	0.09486 (7)	0.0339 (4)	
H1	−0.279883	0.227204	0.119239	0.041*	
C2	−0.2285 (4)	0.1725 (3)	0.04807 (7)	0.0409 (5)	
H2	−0.378344	0.085771	0.039952	0.049*	
C3	−0.0693 (4)	0.2135 (3)	0.01278 (7)	0.0401 (4)	
H3	−0.108425	0.156670	−0.019922	0.048*	
C4	0.1481 (4)	0.3385 (3)	0.02579 (7)	0.0390 (4)	
H4	0.262145	0.367154	0.002180	0.047*	
C5	0.1988 (3)	0.4213 (2)	0.07289 (7)	0.0339 (4)	
H5	0.348200	0.507836	0.081857	0.041*	
C6	0.0934 (3)	0.4768 (2)	0.15689 (6)	0.0311 (4)	
H6A	−0.046325	0.435083	0.176244	0.037*	
H6B	0.104912	0.608820	0.154518	0.037*	
C7	0.3304 (3)	0.4468 (2)	0.18408 (6)	0.0314 (4)	
H7A	0.473101	0.495722	0.166282	0.038*	
H7B	0.324144	0.314736	0.185476	0.038*	
C8	0.3644 (3)	0.5410 (2)	0.23604 (6)	0.0320 (4)	
H8A	0.367090	0.672402	0.234064	0.038*	
H8B	0.219492	0.491783	0.253230	0.038*	
C9	0.5985 (3)	0.5194 (3)	0.26641 (6)	0.0337 (4)	
H9A	0.743876	0.570106	0.249546	0.040*	
H9B	0.596903	0.388158	0.268263	0.040*	
C10	0.6273 (3)	0.6130 (3)	0.31830 (6)	0.0331 (4)	
H10A	0.479346	0.564481	0.334707	0.040*	
H10B	0.632261	0.744601	0.316285	0.040*	
C11	0.8573 (3)	0.5898 (3)	0.34997 (6)	0.0336 (4)	
H11A	1.005506	0.636551	0.333400	0.040*	
H11B	0.851193	0.458422	0.352611	0.040*	
C12	0.8855 (3)	0.6873 (3)	0.40158 (6)	0.0328 (4)	
H12A	0.892594	0.818747	0.398871	0.039*	
H12B	0.736422	0.641239	0.417977	0.039*	
C13	1.1140 (3)	0.6639 (3)	0.43375 (6)	0.0336 (4)	
H13A	1.106417	0.532484	0.436662	0.040*	
H13B	1.263054	0.709292	0.417293	0.040*	
C14	1.1420 (3)	0.7623 (3)	0.48512 (6)	0.0334 (4)	
H14A	1.149548	0.893655	0.482182	0.040*	
H14B	0.992850	0.716892	0.501544	0.040*	
C15	1.3701 (3)	0.7392 (3)	0.51738 (6)	0.0337 (4)	
H15A	1.519340	0.784577	0.500963	0.040*	
H15B	1.362641	0.607804	0.520323	0.040*	
C16	1.3980 (3)	0.8377 (3)	0.56876 (6)	0.0338 (4)	
H16A	1.248828	0.792293	0.585183	0.041*	
H16B	1.405644	0.969037	0.565828	0.041*	
C17	1.6265 (3)	0.8144 (3)	0.60107 (6)	0.0337 (4)	
H17A	1.619189	0.682963	0.603911	0.040*	
H17B	1.775761	0.860093	0.584696	0.040*	
C18	1.6542 (3)	0.9124 (3)	0.65253 (6)	0.0338 (4)	
H18A	1.660006	1.043577	0.649665	0.041*	
H18B	1.505536	0.865875	0.668984	0.041*	
C19	1.8831 (3)	0.8908 (3)	0.68477 (6)	0.0328 (4)	
H19A	1.876876	0.759561	0.687780	0.039*	
H19B	2.031742	0.936616	0.668231	0.039*	
C20	1.9115 (4)	0.9893 (3)	0.73601 (7)	0.0382 (4)	
H20A	1.763700	0.942615	0.752705	0.046*	
H20B	1.916372	1.120340	0.733038	0.046*	
C21	2.1420 (4)	0.9684 (3)	0.76790 (7)	0.0460 (5)	
H21A	2.137427	0.839242	0.771662	0.069*	
H21B	2.147622	1.034676	0.800381	0.069*	
H21C	2.289833	1.017933	0.752238	0.069*	
H1A	0.852 (2)	0.778 (3)	0.0994 (9)	0.069 (8)*	
H1B	0.614 (4)	0.784 (3)	0.1003 (10)	0.081 (10)*	

### 1-Hexadecylpyridin-1-ium bromide monohydrate Atomic displacement parameters (Å^2^)

**Table:**

	*U*^11^	*U*^22^	*U*^33^	*U*^12^	*U*^13^	*U*^23^
Br1	0.02816 (10)	0.03556 (11)	0.03999 (11)	0.00062 (7)	0.00212 (7)	−0.00012 (7)
O1	0.0321 (8)	0.0411 (8)	0.0731 (11)	−0.0018 (7)	0.0078 (7)	−0.0051 (7)
N1	0.0263 (7)	0.0260 (7)	0.0262 (7)	0.0042 (6)	−0.0031 (5)	0.0027 (6)
C1	0.0274 (9)	0.0337 (10)	0.0376 (10)	−0.0011 (7)	0.0003 (7)	0.0061 (8)
C2	0.0374 (10)	0.0383 (10)	0.0388 (10)	−0.0061 (8)	−0.0086 (8)	−0.0011 (8)
C3	0.0520 (12)	0.0367 (10)	0.0293 (9)	0.0095 (9)	−0.0061 (8)	−0.0021 (8)
C4	0.0429 (11)	0.0436 (11)	0.0303 (9)	0.0057 (9)	0.0049 (8)	0.0080 (8)
C5	0.0310 (9)	0.0341 (10)	0.0337 (9)	−0.0019 (7)	0.0016 (7)	0.0074 (7)
C6	0.0327 (9)	0.0313 (9)	0.0278 (9)	0.0067 (7)	−0.0015 (7)	−0.0016 (7)
C7	0.0321 (9)	0.0336 (9)	0.0276 (9)	0.0082 (7)	−0.0020 (7)	−0.0012 (7)
C8	0.0322 (9)	0.0347 (10)	0.0282 (9)	0.0089 (8)	−0.0018 (7)	−0.0035 (7)
C9	0.0325 (9)	0.0383 (10)	0.0294 (9)	0.0109 (8)	−0.0027 (7)	−0.0054 (7)
C10	0.0310 (9)	0.0394 (10)	0.0283 (9)	0.0103 (8)	−0.0011 (7)	−0.0042 (7)
C11	0.0325 (9)	0.0395 (10)	0.0282 (9)	0.0110 (8)	−0.0015 (7)	−0.0046 (7)
C12	0.0318 (9)	0.0388 (10)	0.0277 (9)	0.0107 (8)	−0.0008 (7)	−0.0032 (7)
C13	0.0317 (9)	0.0396 (10)	0.0290 (9)	0.0106 (8)	−0.0011 (7)	−0.0032 (7)
C14	0.0311 (9)	0.0400 (10)	0.0287 (9)	0.0103 (8)	−0.0007 (7)	−0.0037 (7)
C15	0.0319 (9)	0.0404 (10)	0.0285 (9)	0.0111 (8)	−0.0016 (7)	−0.0032 (7)
C16	0.0315 (9)	0.0407 (10)	0.0292 (9)	0.0115 (8)	−0.0008 (7)	−0.0035 (8)
C17	0.0323 (9)	0.0393 (10)	0.0292 (9)	0.0109 (8)	−0.0011 (7)	−0.0032 (7)
C18	0.0320 (9)	0.0402 (10)	0.0292 (9)	0.0108 (8)	−0.0004 (7)	−0.0022 (8)
C19	0.0326 (9)	0.0370 (10)	0.0293 (9)	0.0108 (8)	0.0003 (7)	−0.0014 (7)
C20	0.0370 (10)	0.0482 (11)	0.0294 (9)	0.0123 (9)	0.0001 (8)	−0.0028 (8)
C21	0.0429 (11)	0.0602 (13)	0.0324 (10)	0.0108 (10)	−0.0059 (8)	−0.0013 (9)

### 1-Hexadecylpyridin-1-ium bromide monohydrate Geometric parameters (Å, º)

**Table:**

O1—H1A	0.837 (10)	C11—H11B	0.9900
O1—H1B	0.838 (10)	C12—C13	1.519 (2)
N1—C1	1.337 (2)	C12—H12A	0.9900
N1—C5	1.347 (2)	C12—H12B	0.9900
N1—C6	1.483 (2)	C13—C14	1.519 (2)
C1—C2	1.368 (3)	C13—H13A	0.9900
C1—H1	0.9500	C13—H13B	0.9900
C2—C3	1.376 (3)	C14—C15	1.517 (2)
C2—H2	0.9500	C14—H14A	0.9900
C3—C4	1.379 (3)	C14—H14B	0.9900
C3—H3	0.9500	C15—C16	1.519 (2)
C4—C5	1.369 (3)	C15—H15A	0.9900
C4—H4	0.9500	C15—H15B	0.9900
C5—H5	0.9500	C16—C17	1.520 (2)
C6—C7	1.509 (2)	C16—H16A	0.9900
C6—H6A	0.9900	C16—H16B	0.9900
C6—H6B	0.9900	C17—C18	1.520 (2)
C7—C8	1.519 (2)	C17—H17A	0.9900
C7—H7A	0.9900	C17—H17B	0.9900
C7—H7B	0.9900	C18—C19	1.517 (2)
C8—C9	1.515 (2)	C18—H18A	0.9900
C8—H8A	0.9900	C18—H18B	0.9900
C8—H8B	0.9900	C19—C20	1.516 (2)
C9—C10	1.517 (2)	C19—H19A	0.9900
C9—H9A	0.9900	C19—H19B	0.9900
C9—H9B	0.9900	C20—C21	1.518 (3)
C10—C11	1.518 (2)	C20—H20A	0.9900
C10—H10A	0.9900	C20—H20B	0.9900
C10—H10B	0.9900	C21—H21A	0.9800
C11—C12	1.522 (2)	C21—H21B	0.9800
C11—H11A	0.9900	C21—H21C	0.9800
			
H1A—O1—H1B	104.1 (18)	C13—C12—H12B	108.7
C1—N1—C5	120.59 (15)	C11—C12—H12B	108.7
C1—N1—C6	119.99 (15)	H12A—C12—H12B	107.6
C5—N1—C6	119.42 (14)	C14—C13—C12	113.91 (14)
N1—C1—C2	120.62 (17)	C14—C13—H13A	108.8
N1—C1—H1	119.7	C12—C13—H13A	108.8
C2—C1—H1	119.7	C14—C13—H13B	108.8
C1—C2—C3	119.91 (17)	C12—C13—H13B	108.8
C1—C2—H2	120.0	H13A—C13—H13B	107.7
C3—C2—H2	120.0	C15—C14—C13	114.07 (15)
C2—C3—C4	118.72 (17)	C15—C14—H14A	108.7
C2—C3—H3	120.6	C13—C14—H14A	108.7
C4—C3—H3	120.6	C15—C14—H14B	108.7
C5—C4—C3	119.71 (18)	C13—C14—H14B	108.7
C5—C4—H4	120.1	H14A—C14—H14B	107.6
C3—C4—H4	120.1	C14—C15—C16	114.04 (15)
N1—C5—C4	120.43 (16)	C14—C15—H15A	108.7
N1—C5—H5	119.8	C16—C15—H15A	108.7
C4—C5—H5	119.8	C14—C15—H15B	108.7
N1—C6—C7	113.70 (14)	C16—C15—H15B	108.7
N1—C6—H6A	108.8	H15A—C15—H15B	107.6
C7—C6—H6A	108.8	C15—C16—C17	114.01 (15)
N1—C6—H6B	108.8	C15—C16—H16A	108.8
C7—C6—H6B	108.8	C17—C16—H16A	108.8
H6A—C6—H6B	107.7	C15—C16—H16B	108.8
C6—C7—C8	110.07 (14)	C17—C16—H16B	108.8
C6—C7—H7A	109.6	H16A—C16—H16B	107.6
C8—C7—H7A	109.6	C18—C17—C16	114.06 (15)
C6—C7—H7B	109.6	C18—C17—H17A	108.7
C8—C7—H7B	109.6	C16—C17—H17A	108.7
H7A—C7—H7B	108.2	C18—C17—H17B	108.7
C9—C8—C7	114.14 (14)	C16—C17—H17B	108.7
C9—C8—H8A	108.7	H17A—C17—H17B	107.6
C7—C8—H8A	108.7	C19—C18—C17	114.24 (15)
C9—C8—H8B	108.7	C19—C18—H18A	108.7
C7—C8—H8B	108.7	C17—C18—H18A	108.7
H8A—C8—H8B	107.6	C19—C18—H18B	108.7
C8—C9—C10	113.24 (14)	C17—C18—H18B	108.7
C8—C9—H9A	108.9	H18A—C18—H18B	107.6
C10—C9—H9A	108.9	C20—C19—C18	114.25 (15)
C8—C9—H9B	108.9	C20—C19—H19A	108.7
C10—C9—H9B	108.9	C18—C19—H19A	108.7
H9A—C9—H9B	107.7	C20—C19—H19B	108.7
C9—C10—C11	114.38 (14)	C18—C19—H19B	108.7
C9—C10—H10A	108.7	H19A—C19—H19B	107.6
C11—C10—H10A	108.7	C19—C20—C21	113.96 (16)
C9—C10—H10B	108.7	C19—C20—H20A	108.8
C11—C10—H10B	108.7	C21—C20—H20A	108.8
H10A—C10—H10B	107.6	C19—C20—H20B	108.8
C10—C11—C12	113.63 (14)	C21—C20—H20B	108.8
C10—C11—H11A	108.8	H20A—C20—H20B	107.7
C12—C11—H11A	108.8	C20—C21—H21A	109.5
C10—C11—H11B	108.8	C20—C21—H21B	109.5
C12—C11—H11B	108.8	H21A—C21—H21B	109.5
H11A—C11—H11B	107.7	C20—C21—H21C	109.5
C13—C12—C11	114.11 (14)	H21A—C21—H21C	109.5
C13—C12—H12A	108.7	H21B—C21—H21C	109.5
C11—C12—H12A	108.7		
			
C5—N1—C1—C2	−1.7 (3)	C7—C8—C9—C10	−179.42 (16)
C6—N1—C1—C2	177.39 (16)	C8—C9—C10—C11	178.63 (16)
N1—C1—C2—C3	0.8 (3)	C9—C10—C11—C12	179.00 (16)
C1—C2—C3—C4	0.7 (3)	C10—C11—C12—C13	179.56 (16)
C2—C3—C4—C5	−1.2 (3)	C11—C12—C13—C14	179.66 (16)
C1—N1—C5—C4	1.2 (3)	C12—C13—C14—C15	179.98 (16)
C6—N1—C5—C4	−177.93 (16)	C13—C14—C15—C16	180.00 (16)
C3—C4—C5—N1	0.3 (3)	C14—C15—C16—C17	179.97 (16)
C1—N1—C6—C7	119.68 (18)	C15—C16—C17—C18	−179.82 (16)
C5—N1—C6—C7	−61.2 (2)	C16—C17—C18—C19	−179.52 (16)
N1—C6—C7—C8	−176.85 (14)	C17—C18—C19—C20	179.70 (16)
C6—C7—C8—C9	−179.81 (16)	C18—C19—C20—C21	−179.49 (17)

### 1-Hexadecylpyridin-1-ium bromide monohydrate Hydrogen-bond geometry (Å, º)

**Table:**

*D*—H···*A*	*D*—H	H···*A*	*D*···*A*	*D*—H···*A*
O1—H1*A*···Br1^i^	0.84 (1)	2.49 (1)	3.3254 (14)	177 (2)
O1—H1*B*···Br1	0.84 (1)	2.49 (1)	3.3227 (16)	171 (2)
C1—H1···Br1^ii^	0.95	2.87	3.5890 (17)	133
C3—H3···Br1^iii^	0.95	2.85	3.7797 (18)	168
C4—H4···O1^iv^	0.95	2.59	3.362 (3)	138
C5—H5···O1	0.95	2.28	3.218 (2)	171
C6—H6*B*···Br1	0.99	2.84	3.7702 (18)	156

Symmetry codes: (i) *x*+1, *y*, *z*; (ii) *x*−1, *y*−1, *z*; (iii) −*x*, −*y*+1, −*z*; (iv) −*x*+1, −*y*+1, −*z*.
